# Association of physical activity and sitting with metabolic syndrome and hyperglycemic clamp parameters in adolescents – BRAMS pediatric study

**DOI:** 10.3389/fendo.2023.1191935

**Published:** 2023-06-15

**Authors:** Talita Oliveira Silva, Marina Maintinguer Norde, Ana Carolina Vasques, Mariana Porto Zambom, Maria Angela Reis de Góes Monteiro Antonio, Ana Maria De Bernardi Rodrigues, BRAMS group, Bruno Geloneze, Brazilian Metabolic Syndrome Study investigators

**Affiliations:** ^1^ Laboratory of Diabetes and Metabolism Investigation (LIMED), School of Medical Sciences of the State University of Campinas (FCM-UNICAMP), Campinas, SP, Brazil; ^2^ Department of Pediatrics, FCM-UNICAMP, Campinas, SP, Brazil; ^3^ School of Health and Life Sciences, Nossa Senhora do Patrocinio University, Itu, SP, Brazil

**Keywords:** physical activity, international physical activity questionnaire, sitting, metabolic syndrome, adolescents, hyperglycemic clamp

## Abstract

**Introduction:**

Obesity and metabolic syndrome (MetS) have immediate and long-term consequences on adolescent health and well-being. Among the available treatments for MetS in adolescents, behavioral interventions such as increasing physical activity (PA) are preferred. This study aimed to investigate the association of PA and sitting time with MetS and a complete set of metabolic health parameters.

**Methods:**

Data from the Pediatric Brazilian Metabolic Syndrome Study (BRAMS-P), a cross-sectional multicenter study conducted using a convenience sample of 448 Brazilian adolescents (10y–19y), were used. Sociodemographic and lifestyle information were collected using a standardized questionnaire. Daily PA and sitting time were estimated from the International PA Questionnaire. Anthropometric parameters, body composition, and blood pressure were measured by trained researchers. Blood lipids, uric acid, hepatic enzymes, creatinine, glycated hemoglobin, glucose, and insulin were measured in fasting blood samples, and the Homeostasis Model Assessment for Insulin Resistance was calculated. A subsample of 57 adolescents underwent the hyperglycemic clamp protocol.

**Results:**

The odds for metabolic syndrome were higher among adolescents who spent >8h sitting (OR (95%CI)=2.11 (1.02 – 4.38)), but not in those classified as active (OR (95%CI)=0.98 (0.42 – 2.26)). Adolescents who spent more time sitting had higher BMI, waist circumference, sagittal abdominal diameter, neck circumference, percentage of body fat, and worse blood lipid profile. The insulin sensitivity index was moderately and positively correlated with moderate-to-high PA in minutes per day (rho=0.29; p=0.047).

**Conclusion:**

Time spent sitting was associated with worse metabolic parameters and must be restricted in favor of adolescent health. Regular PA is associated with improved insulin sensitivity and may be encouraged not only in adolescents with obesity or metabolic disorders but also to prevent adverse metabolic outcomes in normal-weight adolescents.

## 1 Introduction

Adolescence is a critical period in human development given the physiological, sociological, psychological, and reproductive maturation that occurs during this stage of life (1). The prevalence of obesity in children and adolescents between 5 and 19 years of age has almost doubled during the last 20 years, reaching 18.4% globally (2), raising concerns about its immediate and long-term consequences on adolescents’ health and well-being (3).

Adolescents with obesity have higher risk of anxiety and depression (4), polycystic ovary syndrome (5), insulin resistance, hypertension and dyslipidemia, many of which share components with the so-called metabolic syndrome (MetS) (6). In addition, when the onset of these metabolic disorders occurs during childhood or adolescence, there is an increased risk for diabetes, cardiovascular diseases, and some types of cancer before the age of 45 years, posing a huge burden upon health systems around the globe (7).

As defined by the International Diabetes federation (IDF), MetS is a cluster of interrelated risk factors for cardiovascular disease and type 2 diabetes, including abdominal obesity, high cholesterol levels, hypertension, and impaired insulin sensitivity, which are defined by anthropometric, blood pressure, and blood biomarkers specific cut-off values depending on adolescent’s age range (8). Along with obesity, the prevalence of MetS is increasing, reaching approximately 35.5 million adolescents worldwide (9).

Among the available treatment options for MetS in children and adolescents, behavioral interventions such as improving dietary quality and adequate physical activity (PA) are prioritized over drugs and surgical therapy (10). In this sense, studies have shown that greater amounts of moderate-to-high intensity PA, objectively measured (accelerometer) and self-reported, are associated with a lower risk for MetS and other cardiometabolic health outcomes in adolescents (11–13). For sedentary behavior, on the other hand, while screen time is a well-known risk factor for MetS, in children and adolescents, as reviewed elsewhere (14), there are conflicting results and recommendations on sitting time (11, 12).

Moreover, most studies have investigated basic outcomes in relation to PA and sedentary behavior, such as body mass index and metabolic syndrome components (11, 14); however, few studies have investigated a complete set of metabolic health biomarkers, which compromises physical activity and sedentary behavior (12). To our knowledge, only one study has assessed the relationship between physical activity and direct measures of insulin sensitivity and beta-cell function using the hyperglycemic-clamp protocol in adolescents (15), and no study has investigated these outcomes in relation to sedentary behaviors. Thus, the present study aimed to investigate the association of moderate-to-high-level physical activity and sitting time with MetS and a complete set of metabolic health outcomes, including the investigation of hyperglycemic clamp parameters in a subsample.

## 2 Materials and methods

### 2.1 Study design

The present study used data from the Pediatric Brazilian Metabolic Syndrome Study (BRAMS-P), a cross-sectional study conducted on a convenience sample of adolescents between 2011 and 2013, which took place in health centers, ambulatories, public schools, and public universities across three Brazilian cities: Campinas, Itu, and Sao Paulo.

Individuals between 10 and 19 years of age were invited to participate and had a body mass index above the 5th percentile, according to the Centers for Disease Control and Prevention growth chart for age and sex (16). Individuals were excluded at the time of data and sample collection, if they were pregnant, or presented with liver disease, nephropathy, hypothyroidism, hyperthyroidism, diabetes mellitus, genetic syndrome diagnosis, and delayed neuropsychomotor development, as well as those who were using either systemic corticosteroids or drugs with hypoglycemic properties.

For the present study, further exclusions were made if individuals had incomplete data to diagnose metabolic syndrome (missing values for any of the following: plasma high-density lipoprotein cholesterol [HDL-c] concentration, fasting glucose, blood pressure, and waist circumference) or did not complete the International Physical Activity Questionnaire (IPAQ).

All participants and their legal guardians were informed of the study protocol, and those who agreed to participate signed an informed consent form. The study protocol was approved by the Committee for Research Ethics of the School of Medical Sciences of UNICAMP (protocol n. 900/2010, CAAE: 0696.0.146.146-10) and is in accordance with the Brazilian law and the ethical principles of Helsinki Declaration.

### 2.2 Clinical evaluation

Data on demographic (age and sex) and socioeconomic (chief-or-the-family educational level, and Brazilian economic classification table) characteristics, as well as on family health history (hypertension, obesity, dyslipidemia, cardiovascular disease and diabetes), smoking habits, alcohol intake, other illicit drug use, supplement use, medicine use, and sleeping habits were collected by trained interviewers using a standardized questionnaire.

Sexual maturity was rated according to Tanner scale (17), which was presented to participants in a reserved room by trained researchers and self-declared, and pubertal development was determined as pre-pubertal (Tanner I), pubertal (Tanner II-IV) and post-pubertal (Tanner V). Further information on BRAMS-p self-assessment method can be found elsewhere (18). Blood pressure was measured using a mercury-based sphygmomanometer with auscultatory approach, following National High Blood Pressure Education Program Working Group on High Blood Pressure in Children and Adolescents recommendations (19).

Additionally, Campinas and Itu centers used a IPAQ-short form applied by trained interviewers adapted to the Brazilian population (20), from which the time spent on moderate and intense PA as well as the time spent on sitting position per day were calculated.

Adolescents were classified as having metabolic syndrome following the IDF criteria (8).

### 2.3 Anthropometric measurements and body composition parameters

Adolescents were asked to wear light clothing and no shoes during all the anthropometric and body composition evaluation. Body weight was measured using a digital scale with capacity for 150 Kg and precision of 0,1 Kg, and height was measured with adolescents standing in an orthostatic position against a wall, using a fixed stadiometer with capacity for 220 cm and precision of 0,1 cm. Body mass index (BMI) was, then, calculated as body weight, in Kg, divided by squared height, in cm. BMI was transformed into z-score using the LMS parameters from the World Health Organization (WHO) BMI-for-age growth chart for boys and girls, and classified as overweight and obesity according to the WHO cut-off points (21).

Waist circumference was measured by trained researchers positioning the tape at the midpoint between the last rib and the iliac crest. Hip circumference was measured positioning the tape at the biggest circumference between the waist and knees while adolescents were at the stand position with feet 30 cm apart (22). The sagittal abdominal diameter was measured using the Holtain-Kahn Abdominal Caliper (Holtain Ltd, Crymych, United Kingdom), at the umbilicus level after a normal exhalation while the subjects were in a supine position with their knees slightly bent on a firm examination table (22). The neck circumference was measured positioning the tape at the midpoint of the neck length (23).

Percentage body fat was estimated using tetrapolar bioimpedance (Biodynamics, model 310, Shoreline, Washington, USA) validated for epidemiological studies (24).

### 2.4 Biochemical markers

Blood samples were collected after a 12-hour overnight fasting, and centrifuged for plasma storage at 80°C. Plasma samples were transported to the UNICAMP Clinical Hospital laboratory, where creatinine, glucose, total cholesterol, HDL-c, low-density lipoprotein cholesterol (LDL-c), triglycerides, uric acid, gamma-glutamil transferase (gamma-GT), aspartate aminotransferase (AST), alanine aminotransferase (ALT), and glycated hemoglobin were measured using standard protocols (25). Insulin plasma levels were measured by enzyme-linked immunosorbent assay kit (EZHI-14K; Millipore; St. Louis, Missouri, USA) at the Laboratory of Diabetes and Metabolism Investigations (LIMED).

The Homeostasis Model Assessment for Insulin Resistance (HOMA-IR) was calculated as the product of the fasting plasma insulin level (in milliunits per liter) and the fasting plasma glucose level (in millimoles per liter), divided by 22.5 (26).

### 2.5 Metabolic syndrome criteria

Metabolic syndrome was defined according to the International Diabetes Federation criteria (27). For adolescents aging 10 to 16 years, the MetS was established whenever high waist circumference was present (> 90^th^ percentile) along with at least two of the following components: high blood pressure (systolic or diastolic blood pressure > 95^th^ percentile); low HDL-c (≤ 40 mg/dL); and high fasting glucose (>100 mg/dL). For adolescents aging more than 16 years, MetS was established when three or more of the following components were present: high waist circumference (≥94 cm for men, and ≥80 cm for women); high blood pressure (systolic blood pressure ≥130 mmHg or diastolic blood pressure ≥ 85 mmHg); low HDL-c (≤ 40 mg/dL for men, and ≤ 50 mg/dL for women); and high fasting glucose (> 100 mg/dL).

### 2.6 Hyperglycemic clamp protocol

Participants underwent a 2-hour hyperglycemic clamp (with blood glucose acutely raised and maintained at approximately 225 mg/dL; to convert to millimoles per liter, multiply by 0.0555) according to the protocol previously described by Arslanian (28).The insulin sensitivity index (ISI) was calculated as the mean exogenous glucose infusion rate from 60 to 120 minutes of the clamp protocol, adjusted for urinary glucose excretion (subtraction), divided by the mean insulin concentration of the period, and it was then corrected for lean body mass (29). The Disposition Index (DI), which represents the beta-cell function relative to insulin sensitivity, was calculated as the product of ISI vs. the area under the curve of the first phase of the insulin secretory rate (30).

### 2.7 Statistical analysis

Continuous variables were tested for normality using the Kolmogorov-Smirnov test and, as the vast majority did not have satisfactory adhesion to normal distribution, results are presented as median (min-max). Categorical variables are presented in absolute and relative frequency.

To compare means between adolescents with and without the metabolic syndrome, the Mann-Whitney test was applied. The chi-squared test was applied to compare frequencies between different metabolic syndrome status.

To check for the correlation between time spent on moderate to high intensity PA, as well as time spent sitting per day, and metabolic parameters the Spearman’s coefficient was used, adjusted for confounding variables. To estimate the odds for metabolic syndrome in adolescents that referred more than 60 minutes per day of moderate to high intensity PA, as well as those that referred more than 8 hours per day of sitting, a multiple logistic regression was used, adjusted for confounding variables.

The confounding factors used were: age (years), sex (dichotomous), smoking status (yes/no to “have you smoked 100 cigarettes or more during your whole life?”), alcohol intake (yes/no to “Have you drink one dose or more of alcoholic beverage the past month?”), puberal status, medicine use (yes/no), sleep (in hours, for the correlation coefficient test, and > 8 hours/night in the logistic regression). Time spent sitting and time spent on moderate to high intensity PA were also added as confounding factors of each other’s exposure.

All analysis were conducted using Stata SE software, version 17.0 (StataCorp LLC, Texas, EUA).

## 3 Results

**Figure 1:**
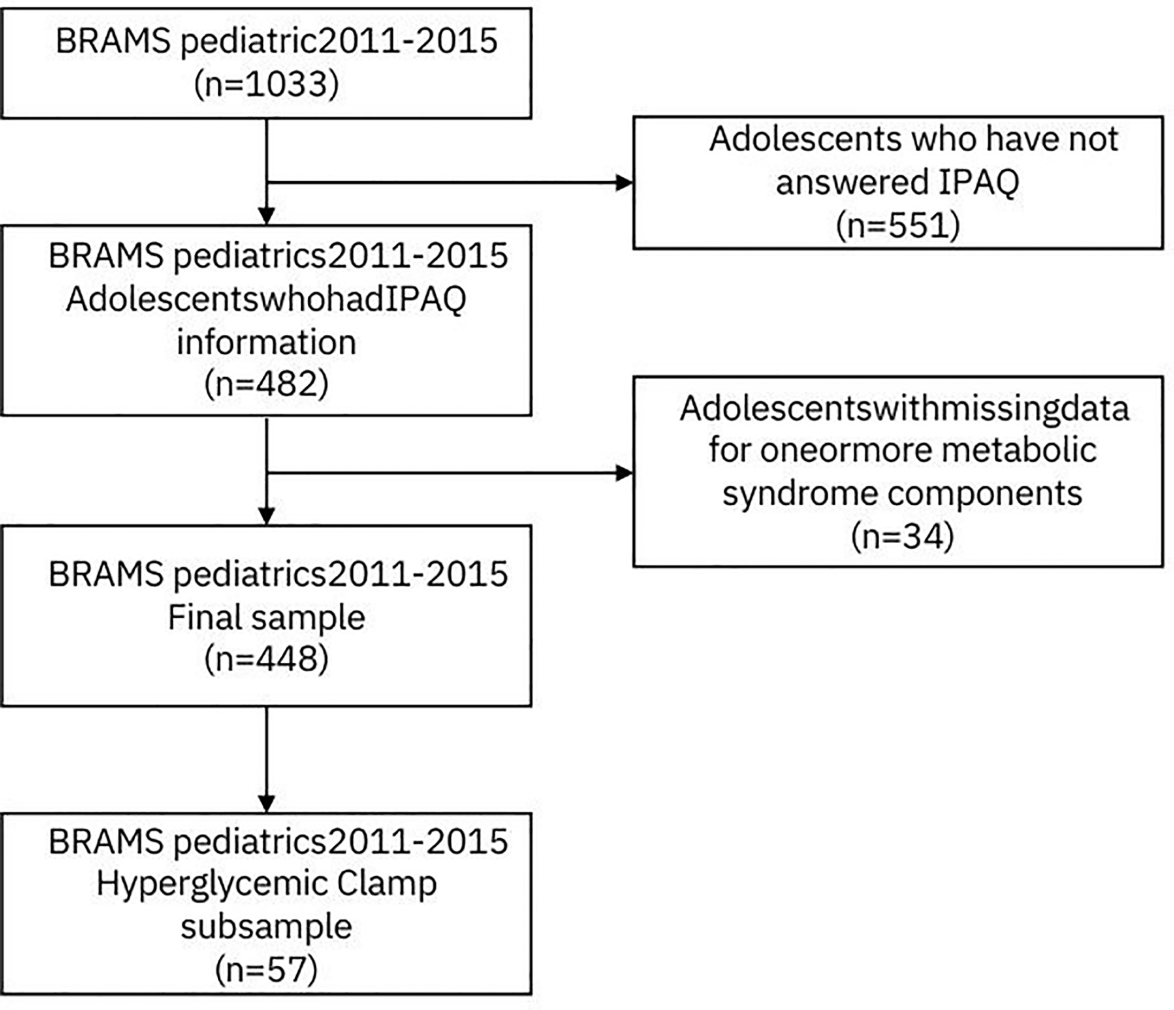
Flowchart on inclusion and exclusion criteria, BRAMS pediatrics, 2011-2015. IPAQ, International Physical Activity Questionnaire.

After applying the exclusion criteria, the final sample of the present study comprised 448 adolescents and a subsample of 57 individuals who participated in the hyperglycemic clamp protocol (**Figure 1**).

There was a balance between males and females, with the majority of the sample classified as pubertal, with a median age of 14 years (10 to 19 years), median time spent in moderate-to-high level PA of 24 min per day (varying from 0 to 509 min), and median time spent sitting of 7 h per day (varying from 0.1 to 18 h) (**Table 1**).

**Table 1:** Sociodemographic and lifestyle characteristics of the total sample, and across metabolic syndrome status, BRAMS pediatrics, 2011-2015.

Characteristics	Total sample (n=448) N (%)/median (min – max)	Metabolic syndrome		P value
		No (n=408) N (%)/median (min – max)	Yes (n=40) N (%)/median (min – max)	
**Sex**				0.028
Female	199 (44)	233 (57)	16 (40)	
Male	249 (56)	175 (43)	24 (60)	
**Age (years)**	14 (10 – 19)	14 (10 – 19)	16 (10 – 19)	0.022
**Puberal status**				0.460
Prepuberal	31 (7)	27 (7)	4 (11)	
Puberal	310 (69)	285 (70)	25 (63)	
Post-puberal	106 (24)	95 (23)	11 (26)	
Nutritional status*				<0.001
Underweight	3 (1)	3 (1)	0 (0)	
Normal weight	165 (37)	165 (40)	0 (0)	
Overweight	108 (24)	107 (26)	1 (3)	
Obesity	172 (38)	133 (33)	39 (97)	
**Smoking status**				0.008
≥ 1 cigarette per month	2 (1)	0 (0)	2 (5)	
< 1 cigarette per month	446 (99)	408 (100)	38 (95)	
**Alcohol use**				0.265
≥ 1 dose per month	41 (9)	39 (10)	2 (5)	
< 1 dose per month	407 (91)	369 (90)	38 (95)	
**Sleep**				0.268
Sufficient (> 8 hours/day)	288 (65)	265 (65)	23 (59)	
Insufficient (< 8 hours/day)	157 (35)	141 (35)	16 (41)	
**Medicine use**				0.001
No	433 (98)	399 (99)	34 (87)	
Yes	10 (2)	5 (1)	5 (13)	
**Moderate-to-high level physical activity (min/day)**	24 (0 – 509)	24 (0 – 508)	36 (0 – 411)	0.350
**Time spent sitting (hours/day)**	7.0 (0.1 – 18.0)	7.0 (0.1 – 18.0)	8.1 (2.3 – 18)	0.070

Continuous variables are presented as median (min-max), and categorical variables are presented in absolute (relative) frequency. To compare means between groups, the Mann-Whitney test was applied, and, to compare frequencies, the chi-squared test was used, or *Fisher’s exact test. P values<0.05 were considered statistically significant.

Comparisons between adolescents with (n=38) and without (n=410) metabolic syndrome showed that those with metabolic syndrome were more frequently male and referred to smoking habits and medication use more frequently than those without metabolic syndrome (**Table 1**). Among adolescents with at least one of metabolic syndrome components, 2% had high plasma glucose (n=9), 13% had high blood pressure (n=58), 42% had high waist circumference (n=189), and 47% had low HDL-c (n=212) (**Supplemental Figure S1** shows a Vann’s diagram for intersection between metabolic syndrome components in the total sample). Adolescents with metabolic syndrome were older, had worse anthropometric parameters, higher systolic and diastolic blood pressure, higher plasma cholesterol, triglyceride, uric acid, gamma-GT, ALT, insulin, and HOMA-IR, and lower HDL-c levels than those who did not (**Tables 1**, **2**). Additionally, in the subsample that undertook the hyperglycemic clamp protocol, adolescents with metabolic syndrome had a lower glucose infusion rate, ISI, and DI than those without metabolic syndrome (**Table 3**).

**Table 2:** Anthropometric parameters, biochemical indicators, and blood pressure of the total sample, and across metabolic syndrome status, BRAMS pediatrics, 2011-2015.

Characteristics	Total sample (n=448) Median (min-max)	Metabolic syndrome		P value
		No (n=410) Median (min-max)	Yes (n=38) Median (min-max)	
Anthropometry and body composition				
BMI (z-score)	1.5 (-3.0 – 4.5)	1.4 (-3 - 4)	3 (1.7 - 4.5)	<0.001
Waist circumference (cm)	83 (49 – 139)	80 (49 - 136)	104 (86 - 139)	<0.001
Waist-to-hip ratio	0.85 (0.52 – 0.68)	0.8 (0.5 - 1.9)	0.9 (0.8 - 1.1)	<0.001
Sagittal abdominal diameter (cm)	17.5 (10.5 – 33.9)	17 (11 - 29)	23 (17 - 34)	<0.001
Neck circumference (cm)	33.5 (25.5 – 46.0)	33 (26 - 45)	39 (32 - 46)	<0.001
Body fat (%)	28.5 (4.9 – 67.8)	28 (5 - 68)	37 (19 - 49)	<0.001
Biochemical indicators				
Total cholesterol (mg/dL)	157 (67 – 286)	156 (90 - 286)	169 (67 - 234)	0.001
HDL-c (mg/dL)	46 (24 – 101)	47 (24 - 101)	36 (27 - 56)	<0.001
LDL-c (mg/dL)	92 (26 – 223)	91 (37 - 223)	95 (26 - 168)	0.054
Triglycerides (mg/dL)	73 (12 – 358)	71 (12 - 233)	156 (45 - 358)	<0.001
Uric acid (mg/dL)	4.7 (0.9 – 10.0)	4.6 (0.9 - 10)	6.2 (3.8 - 9.1)	<0.001
Gamma-GT (U/L)	17 (4 – 131)	17 (4 - 131)	22 (10 - 50)	<0.001
AST (U/L)	20 (9 – 61)	20 (9 - 61)	20 (15 - 34)	0.644
ALT (U/L)	15 (5 – 151)	15 (5 - 151)	19 (9 - 68)	<0.001
HbA1c (%)	5.4 (3.4 – 6.5)	5.4 (3.4 - 6.5)	5.4 (4 - 6.1)	0.318
Glucose (mg/dl)	81 (46 – 110)	81 (46 - 110)	83 (56 - 102)	0.151
Insulin (mU/L)	12.3 (1.4 – 64.7)	12 (1 - 65)	23 (3 - 57)	<0.001
HOMA-IR	2.4 (0.3 – 14.2)	2.2 (0.3 - 12.6)	4.3 (0.6 - 14.2)	<0.001
Blood pressure				
Systolic (mmHg)	110 (75 – 170)	110 (75 - 150)	124 (90 - 170)	<0.001
Diastolic (mmHg)	70 (50 – 110)	70 (50 - 100)	80 (50 - 110)	<0.001

ALT, Alanine aminotransferase; AST, Aspartate aminotransferase; BMI, Body mass index; Gamma-GT, gamma-glutamil transferase; HDL-c, high density lipoprotein cholesterol; HOMA-IR, homeostasis assessment model for insulin resistance; LDL-c, Low density lipoprotein cholesterol. Continuous variables are presented in median (min –max). Means comparison were conducted using the Mann-Whitney’s test. P values <0.05 were considered statistically significant.

**Table 3:** Characterization of the hyperglycemic clamp subsample, and across metabolic syndrome status, BRAMS pediatrics, 2011-2015.

Characteristics	Total subsample (n=57) N (%)/median (min – max)	Metabolic syndrome		P value
		Total subsample (n=57) N (%)/median (min – max)	Yes (n=10) N (%)/median (min – max)	
**Sex**				0.730
Female	28 (49)	24 (51)	4 (40)	
Male	29 (51)	23 (49)	6 (60)	
**Age (years)**	14 (10 – 18)	14 (10 – 18)	14.5 (11 – 18)	0.505
**Puberal status**				0.112
Prepuberal	1 (2)	0 (0)	1 (10)	
Puberal	30 (52)	24 (51)	6 (60)	
Post-puberal	26 (46)	23 (49)	3 (30)	
Nutritional status*				0.036
Underweight	7 (12)	7 (15)	0 (0)	
Normal weight	14 (25)	14 (30)	0 (0)	
Overweight	36 (63)	26 (55)	10 (100)	
Obesity				
**Alcohol use**				0.574
≥ 1 dose per month	5 (9)	5 (11)	0 (0)	
< 1 dose per month	52 (91)	42 (89)	10 (100)	
**Sleep**				0.041
Sufficient (> 8 hours/day)	29 (51)	27 (57)	2 (20)	
Insufficient (< 8 hours/day)	28 (49)	20 (43)	8 (80)	
**Medicine use**				1.000
No	53 (96)	43 (96)	10 (100)	
Yes	2 (4)	2 (4)	0 (0)	
**Moderate-to-high level physical activity (min/day)**	26 (0 – 304)	29 (0 – 304)	6 (0 – 61)	0.051
**Time spent sitting (hours/day)**	8 (0 – 16)	8 (0 – 16)	9 (5 – 14)	0.204
**Glucose infusion rate (mg)**	7 (2 – 18)	7 (3 – 18)	5 (2 – 12)	0.019
**Insulin sensitivity index**	0.05 (0.01 – 0.19)	0.05 (0.01 – 0.19)	0.02 (0.01 – 0.11)	0.011
**Disposition index**	515 (42 – 2298)	570 (42 – 2298)	255 (54 – 934)	0.034

Continuous variables are presented as median (min-max), and categorical variables are presented in absolute (relative) frequency. To compare means between groups, the Mann-Whitney test was applied, and, to compare frequencies, the Fisher’s exact test was used. P values<0.05 were considered statistically significant.

The odds for metabolic syndrome were higher among adolescents who spent more than 8 hours per day sitting, but not in those who spent more than 60 minutes a day of moderate-to-high PA (**Table 4**).

**Table 4:** Odds ratio for metabolic sydrome across physical activity and sitting categories in adolescents (n=448), BRAMS pediatrics, 2011-2015.

PA and sitting categories	Metabolic syndrome	
	OR (IC 95%)	Adjusted OR (IC 95%)
≥ 60 min/day of moderate-to-high PA (n=109)	1,20 (0,58 – 2,49)	0,98 (0,42 – 2,26)
≥ 8hours/day sitting (n=191)	1,93 (1,01 – 3,73)	2,11 (1,02 – 4,38)

PA, Physical activity. Odds ratio for metabolic syndrome estimated from a multiple logistic regression, ajdusted for age (years), sex (dichotomous), smoking status (yes/no), alcohol intake (yes/no), puberal status, medicine use (yes/no), and sufficient sleep (yes/no). Time spent sitting and time spent on moderate to high intensity physical activity were treated as confounding factors of each other’s exposure. Odds ratio with 95% confidence intervals that do not contain the number 1 were considered statistically significant.

**Figure 2:**
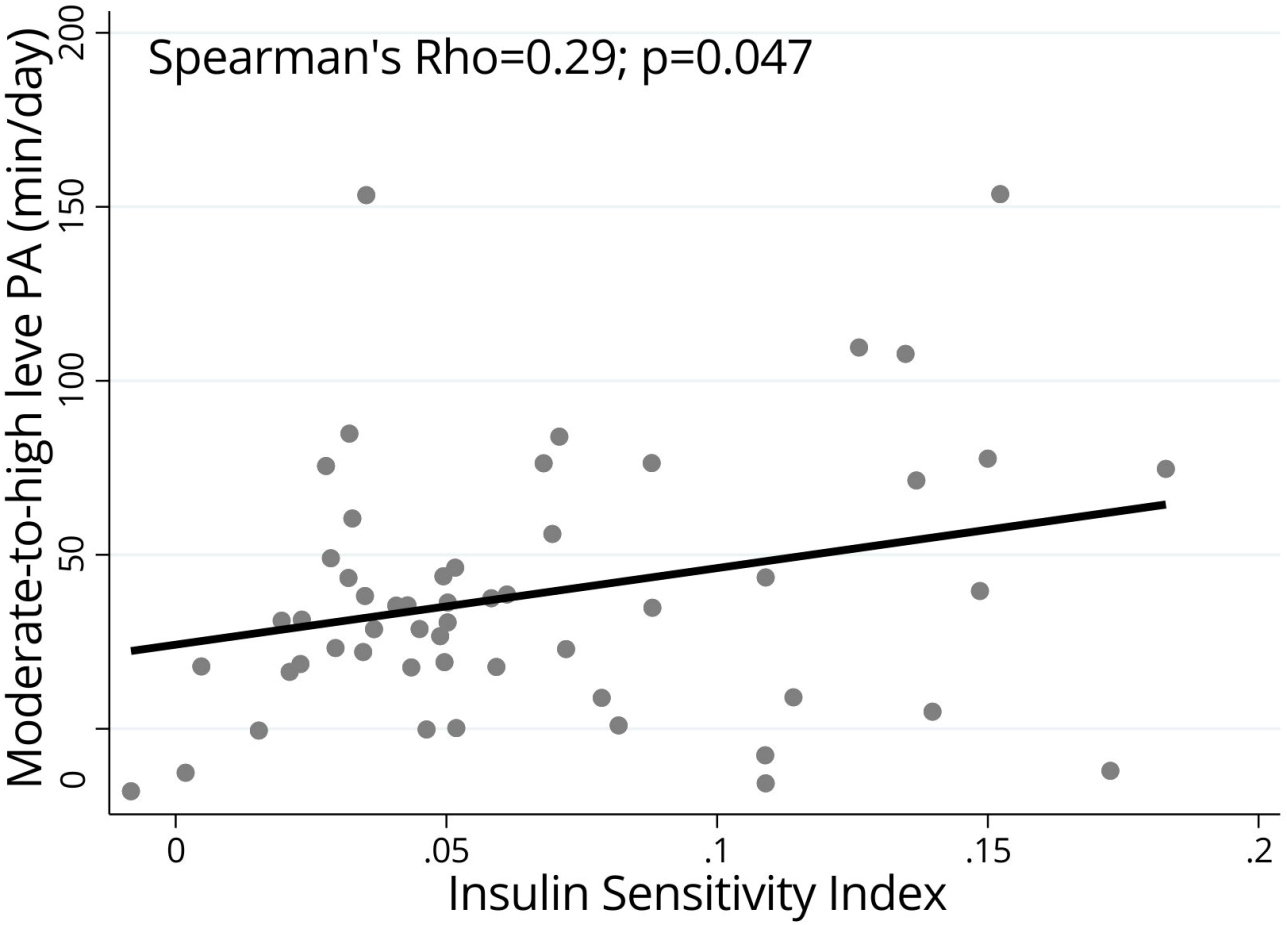
Correlation between the hyperglycemic clamp Insulin Sensitivity Index and time spent in moderate-to-high level physical activity (min/day) fitted values in adolescents (n=57), BRAMS pediatric, 2011-2015. PA, physical activity; Rho - Spearman’s correlation coefficient adjusted for age, sex, smoking status, alcohol intake, puberal status, medicine use, sleep, and time spent sitting.

Adolescents who spent more time sitting had higher BMI, waist circumference, sagittal abdominal diameter, neck circumference, percentage body fat, plasma LDL-c, and triglycerides as well as lower HDL-c, while none of these correlations were found for time spent in moderate-to-high PA (**Table 5**). Among the investigated parameters from the hyperglycemic clamp protocol, ISI had a moderate and positive correlation with moderate-to-high PA, in minutes per day, as shown in **Figure 2**. For the subsample that participated in the hyperglycemic clamp protocol, adolescents who had more moderate-to-high PA daily had lower BMI (rho=-0.31; p=0.031), higher plasma HDL-c (rho=0.35; p=0.016), lower plasma triglyceride levels (rho=-0.32; p=0.027), and lower plasma insulin levels (rho=-0.30; p=0.038), whereas the time spent sitting had no statistically significant correlation with any of the metabolic parameters.

**Table 5:** Correlation between time spent on physical activity and sitting, and metabolic parameters in adolescents, BRAMS pediatrics, 2011-2015.

Metabolic parameters	Sitting (hours/day)	Moderate-to-hight level Physical activity (min/day)
	Rho	Rho
Anthropometry and body composition		
BMI (z-score)	0,15*	-0,04
Waist circumference (cm)	0,16*	-0,01
Waist-to-hip ratio	0,09	-0,03
Sagittal abdominal diameter (cm)	0,12*	-0,03
Neck circumference (cm)	0,11*	|<0,01|
Body fat (%)	0,13*	-0,07
Biochemical indicators		
Total cholesterol (mg/dL)	0,07	|<0,01|
HDL-c (mg/dL)	-0,10*	0,03
LDL-c (mg/dL)	0,12*	-0,02
Triglycerides (mg/dL)	0,10*	0.02
Uric acid (mg/dL)	0,06	0,03
Gamma-GT (U/L)	0,05	-0,09
AST (U/L)	-0,06	0,03
ALT (U/L)	0,01	-0,06
HbA1c (%)	-0,01	0,05
Glucose (mg/dl)	0,09	0,18*
Insulin (mU/L)	0,06	-0,05
HOMA-IR	0,06	-0,01
Blood pressure		
Sistolic (mmHg)	0,05	0,01
Diastolic (mmHg)	0,03	-0,02

ALT, Alanine aminotransferase; AST, Aspartate aminotransferase; BMI, Body mass index; Gamma-GT, gamma-glutamil transferase; HDL-c, high density lipoprotein cholesterol; HOMA-IR, homeostasis assessment model for insulin resistance; LDL-c, Low density lipoprotein cholesterol. Correlation was estimated by Spearman’s coefficient, adjusted for age (years), sex (dichotomous), smoking status (yes/no), alcohol intake (yes/no), puberal status, medicine use (yes/no), and sleep (hours/night). Time spent sitting and time spent on moderate to high intensity physical activity were treated as confounding factors of each other’s exposure. *P values <0.05 were considered statistically significant.

## 4 Discussion

The present study showed that adolescents who spent more time sitting had higher odds for MetS, higher BMI, waist circumference, sagittal abdominal diameter, neck circumference, percentage of body fat, plasma LDL-c, and triglycerides, as well as lower HDL-c. For the subsample from the hyperglycemic clamp protocol, on the other hand, those who spent a greater amount of time on moderate-to-high-level PA had higher insulin sensitivity, as measured by the ISI.

The results regarding the relationship between sitting time and the odds of MetS and its components are controversial. Bae et al., for instance, found in a representative sample of Korean adolescents (12y – 18y), that for each additional hour in daily sitting time, the odds of having at least one MetS component increased by 2% (31). Similarly, Sisson et al. found that daily sitting time was correlated with HOMA-IR in a representative sample of adolescents in the United States (32). Yin and colleagues, on the other hand, besides reporting a relation between sitting time and higher odds for abdominal obesity in a school-based sample of Chinese children and adolescents (6y – 14y), found no association between sitting time and the odds for MetS (33). Similarly, Oliveira and colleagues found that sitting time was not associated with obesity indicators or blood pressure in a Brazilian sample of 6264 adolescents (14y – 19y) (34).

A plausible theory for these controversial results lies in the different activities that compose the amount of sitting time in each study. Sitting time can be subdivided into screen time (TV, computer, and video game), educational activities (homework, classrooms, reading), and others (12). Of these subcategories, strong evidence points to screen time as an important risk factor for MetS in adolescents (14), whereas there is no evidence of harm related to other kinds of sitting activities.

Evidences are, on the other hand, concordant about the effect of moderate-to-high level PA on metabolic health (11–13). The results presented here are in accordance with the work published by Lee et al., who used the hyperglycemic clamp protocol to check for improvements in insulin sensitivity after aerobic and resistance exercise interventions in 43 adolescent boys (12y 0 18y) and showed that increasing moderate-to-high level PA is effective in reducing abdominal adiposity, hepatic lipid accumulation, and, therefore, insulin sensitivity (15). Similarly, in the present study, adolescents that referred more time in moderate-to-high level PA had lower BMI, lower plasma triglyceride levels, and higher insulin sensitivity.

There is biological plausibility for these results. Among other beneficial effects, moderate-to-high levels of PA increase energy expenditure by triggering fatty acid and carbohydrate uptake and oxidation in skeletal muscles, as well as by increasing mitochondrial biogenesis (35). In addition, regular physical exercise decreases systemic low-grade inflammation and modulates the gut microbiome favoring lipid and glucose metabolism, short-chain fatty acids uptake, and secretion of gut hormones with insulin sensitizing effects (36). Ultimately, PA improves insulin sensitivity, as body adiposity, inflammation and disruptive glucose metabolism are critical nodes of insulin resistance pathophysiology (37).

The apparent inconsistency between the results found in total sample compared to that found in the subsample that participated in the hyperglycemic clamp protocol are, in fact, mostly due to the higher sensitivity of hyperglycemic clamp protocol to capture insulin resistance and beta-cell function compared to fasting plasma insulin and glucose levels. Moreover, effects of sitting time were not detected in the aforementioned subsample, and this was probably due to small sample size and, accordingly, lower statistical power.

Some limitations of the present study must be acknowledged. First, even though IPAQ-short form has been previously validated for the Brazilian population (20), self-reported physical activity may be subject to memory bias. Considering that the hyperglycemic clamp protocol is a direct measurement of insulin sensitivity and secretion, an objective measurement of physical activity and sedentary behavior, such as information collected with accelerometers, would improve the precision of the correlation estimation between the two variables. Another limitation is related to the cross-sectional design of the present study, which precludes causal inference and raises concerns about possible reverse causation effects that confuse the results. Reverse causation is a common issue in cross-sectional studies with PA because, on one hand, individuals with overweight, obesity, or metabolic disorders are more likely to have just initiated regular exercise, underestimating the beneficial effects of PA, and, on the other hand, individuals with more serious health issues may be more likely to become inactive, overestimating the protective effects of PA (38).

The present study had several strengths. First, the detailed assessment of metabolic parameters in a large sample of adolescents is rarely found in the literature and allows further investigations on the association of lifestyle and sensitive markers of metabolic disorders, which was extended to a subsample of individuals who participated in the hyperglycemic clamp protocol, a gold standard for insulin secretion evaluation, and a direct measurement of insulin sensitivity (28). Statistical correction for pubertal status and sleep was an important asset, as these factors are well-known confounders, as shown by previous studies with the BRAMS-P dataset using the hyperglycemic clamp protocol (30, 39). Another advantage of the present study was to use the time spent sitting and time spent on moderate-to high-intensity physical activity as confounding factors of each other’s exposure, which favors the interpretation of the results.

In conclusion, independently of the time invested in moderate-to-high-level PA daily, the time spent sitting must be restricted in favor of adolescents’ metabolic health. While our study point to an increase in MetS odds in adolescents that spend more than 8-hours sitting, further studies are needed to investigate the optimum recommendations for sitting and resting time in children and adolescents, standardizing this cut-off point across countries and investigating if the type of activity carried out during this sitting time (e.g.: studying, reading, watching TV) have different impacts in human health. Efforts to fight sedentary behavior in children and adolescents are urgent, especially considering that in 2018, 37% of adolescents globally were sedentary (more than three hours of sitting daily outside school) (40), and this prevalence has rapidly increased, according to recent studies (41), caused by the COVID-19 pandemic. In addition, the World Health Organization recommendations on regular PA are reinforced here to improve insulin sensitivity not only in adolescents with obesity or metabolic disorders but also to prevent adverse metabolic outcomes in normal-weight adolescents (42).

## Data availability statement

The raw data supporting the conclusions of this article will be made available by the authors, without undue reservation.

## Ethics statement

The studies involving human participants were reviewed and approved by Committee for Research Ethics of the School of Medical Sciences of UNICAMP (protocol n. 900/2010, CAAE: 0696.0.146.146-10). Written informed consent to participate in this study was provided by the participants’ legal guardian/next of kin.

## Author contributions

TS, MN and BG contributed to the conception and design of the study. MZ, MA, AR, AV, and BG contributed with data collection. TS and MN conducted statistical analysis and wrote the first draft of the manuscript. All authors contributed to the article and approved the submitted version.
