# Supplementary material for: Association of physical activity and sitting with metabolic syndrome and hyperglycemic clamp parameters in adolescents – BRAMS pediatric study
Source: Front Endocrinol (Lausanne). 2023 Jun 15;14:1191935. doi: 10.3389/fendo.2023.1191935 (PMC10310946; doi:10.3389/fendo.2023.1191935)
Supplement: Supplementary file 1 [file Table_1.docx]

Association of physical activity and sitting with metabolic syndrome and hyperglycemic clamp parameters in adolescents – BRAMS pediatric study

**Talita Oliveira Silva ^1†^, Marina Maintinguer Norde^1†^, Ana Carolina Vasques ^1^, Mariana Porto Zambom^2^, Maria Angela Reis de Góes Monteiro Antonio^2^, Ana Maria De Bernardi Rodrigues^3^, BRAMS group, Bruno Geloneze ^1^* on behaf of the Brazilian Metabolic Syndrome Study investigators**

^1^Laboratory of Diabetes and Metabolism Investigation (LIMED), School of Medical Sciences of the State University of Campinas (FCM-UNICAMP), Campinas, SP, Brazil.

^2^Department of Pediatrics, FCM-UNICAMP, Campinas, SP, Brazil.

^3^School of Health and Life Sciences, Nossa Senhora do Patrocinio University, Itu, SP, Brazil.

^†^ These authors share first authorship

**SUPPLEMENTAL MATERIAL**

**
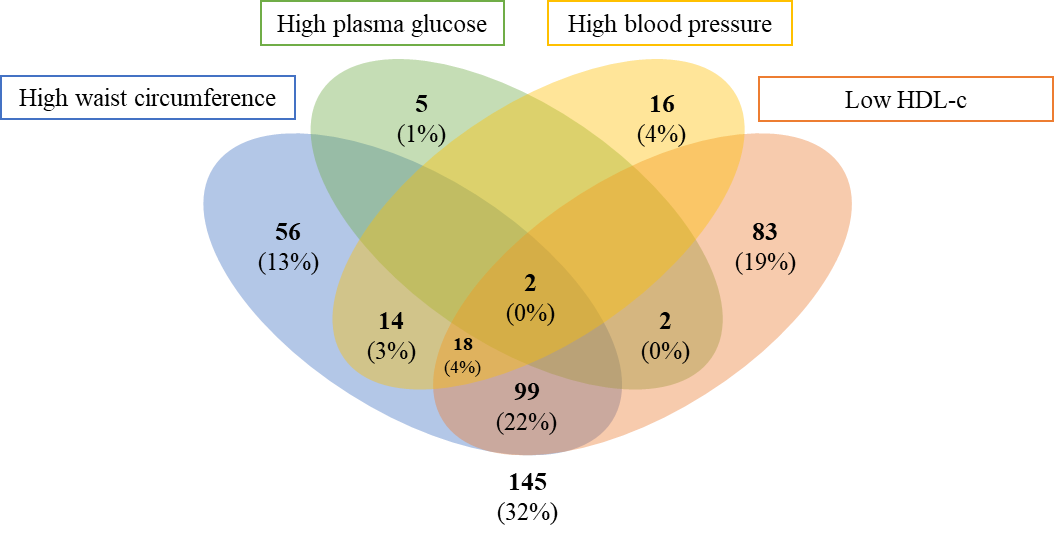
**

**Figure S1**. Venn’s diagram for metabolic syndrome componentes distribution in the study sample (n=448), BRAMS-pediatric, 2011-2015.
